# drSMALL: Database for disease resistance-shaping small molecules derived from the plant microbiome

**DOI:** 10.1007/s44297-025-00042-7

**Published:** 2025-01-21

**Authors:** Rui Cheng, Tingli Ke, Fangze Gui, Junnan Li, Xiaoyan Zhang, Juan Ignacio Vílchez, Haruna Matsumoto

**Affiliations:** 1 Institute of Pesticide and Environmental Toxicology, College of Agriculture and Biotechnology, Zhejiang University, Hangzhou, 310058 China; 2 State Key Laboratory of Rice Biology and Breeding, Ministry of Agricultural and Rural Affairs Laboratory of Molecular Biology of Crop Pathogens and Insects, Zhejiang University, Hangzhou, 55 310058 China; 3 Zhejiang Design Institute of Water Conservancy and Hydro-electric Power Co., Ltd, Hangzhou, 310002 China; 4 Instituto de Tecnologia Química e Biológica (ITQB)-NOVA, iPlantMicro Lab. Oeiras, Lisboa, Oeiras 2780-157 Portugal

**Keywords:** Database, Microbiome, Pathogens, Small molecules, Disease resistance-shaping

## Abstract

Recent evidence highlights the potential of the plant microbiota to increase host plant disease resistance through the production of bioactive small molecules. However, the absence of comprehensive platforms for rapid access to this information hampers progress in the field. To address this gap, we developed the Disease Resistance-Shaping Small Molecules Database (drSMALL), a freely accessible and continuously updated resource that compiles profiles of microbial small molecules, which were experimentally evidenced to be associated with host disease resistance. drSMALL interlinks detailed information on microbial species, the small molecules they produce, host plants, and targeted pathogens, facilitating streamlined access to essential data. This initiative aims to advance the understanding of small molecules in disease resistance, filling a critical gap in data accessibility while fostering deeper exploration of sustainable agricultural practices. By leveraging the natural capabilities of plant microbiomes, drSMALL seeks to support innovative strategies for enhancing crop health and resilience against diseases.

## Introduction

The current global population has exceeded 8 billion, and such a large population base, combined with the continually increasing number, exerts significant pressure on global agricultural production systems. The issue of food security has gradually attracted worldwide attention. Food security is closely linked to sustainable agricultural development, which requires ensuring both crop health and environmental sustainability [1, 2]. However, current pest and disease management methods for crops rely primarily on chemical control. The improper use of agrochemicals may lead to contamination of crops and the environment, disrupting the ecological balance of nature. Frequent and excessive use of the synthetic agrochemicals, in particular, can induce resistance to these agrochemicals [3], thereby diminishing the effectiveness of disease control measures [4]. As a promising alternative in modern agricultural practices, the regulation of host plant resistance is regarded as one of the most environmentally friendly methods for disease management. Recent studies have indicated that the symbiotic microbiota possesses the potential to positively modulate the disease resistance of host plants [4–7], which is linked to their robust ability to produce secondary metabolites. Nevertheless, owing to the diversity of symbiotic microbiota and host plants, along with the complexity of small molecule functions, contemporary artificial intelligence faces challenges in making accurate predictions. In related fields, substantial time is often required for the retrieval of critical information, yet a platform that provides efficient and rapid access to such data remains notably absent.

Here, we present the development of a novel database, drSMALL (www.meclpmi.com/drSMALL/), which offers a centralized web platform for manually curated, evidence-based datasets of microbial-produced small chemical molecules meticulously extracted from the scientific literature. drSMALL contains information on microbial species capable of producing small molecules. In addition, the database provides data on the small molecules associated with these microbes, information on the host plants of the microbes, and details on the pathogens or diseases targeted by these small molecules. drSMALL encompasses a broad range of data, with host plants spanning various families, including Poaceae [4, 6, 8–20], Solanaceae [8–10, 14, 17–19, 21–25], Piperaceae [9, 22, 25], Cucurbitaceae [17, 21, 22, 25, 26], Brassicaceae [21, 22, 27–31], Rutaceae [15], Fabaceae [10, 21, 23, 27, 29, 32], and several other plant families [10, 33–36]. To the best of our knowledge, drSMALL is the first resource platform to systematically provide information on microbial small molecules in the context of disease resistance through a web-based interface. This platform enables users to easily browse and query data related to small molecules and their role in shaping disease resistance. drSMALL is a continuously updated database and open-access platform, with any new relevant research findings promptly incorporated. Researchers with the latest advancements in this field are also encouraged to contact us to contribute additional data. Through continuous expansion and refinement, drSMALL aims to assist researchers focused on microbial small molecules and their role in disease resistance modulation, as well as the broader scientific community, in gaining a deeper understanding of the roles and functions of these molecules in the complex interplay among host plants, their symbiotic microbiota and pathogens. Furthermore, the database seeks to promote future research and explore new directions in this field.

## Results

### Web interface and usage of drSMALL

**Fig. 1:**
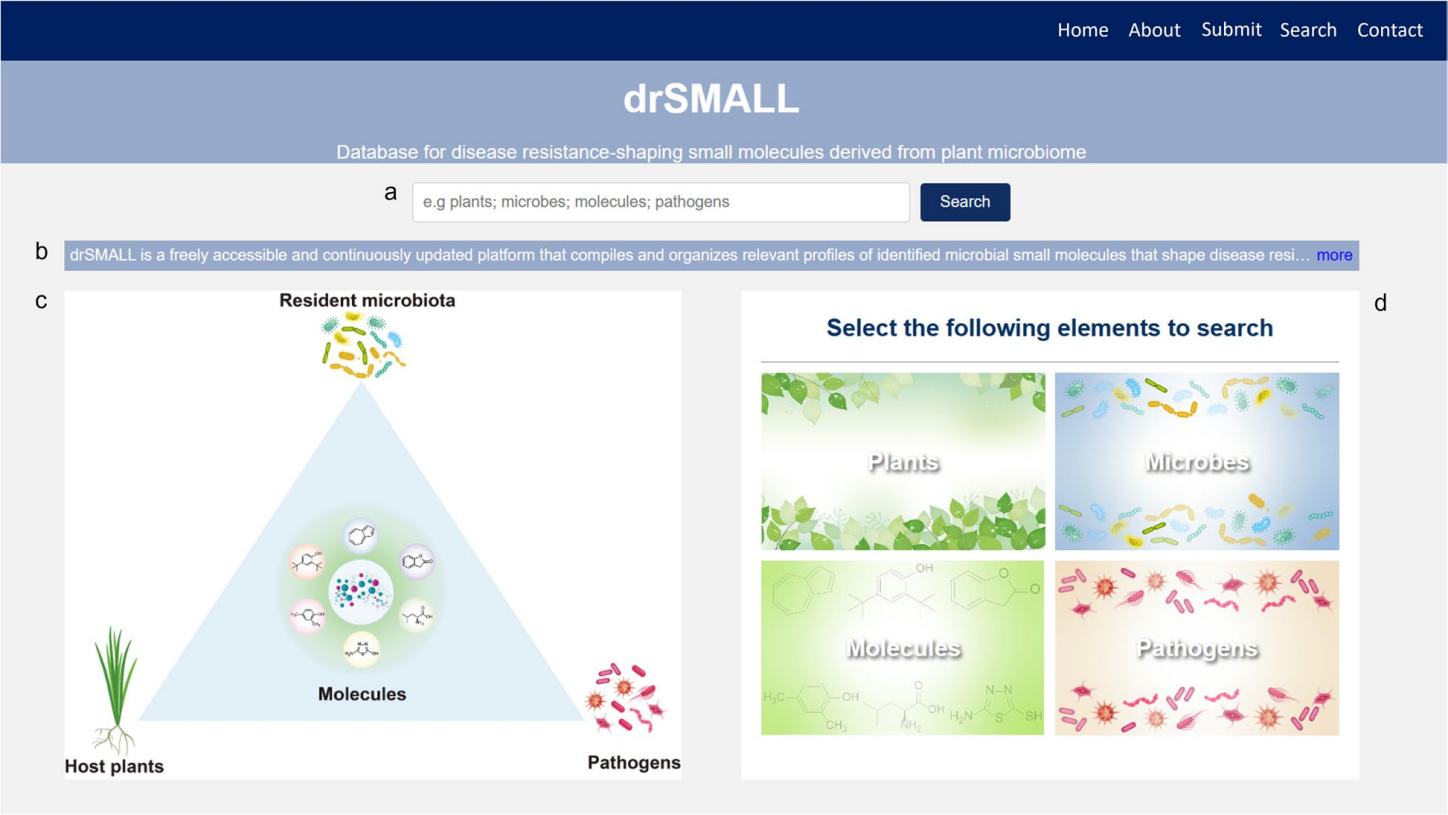
Homepage user interface of drSMALL. **a** The search box allows users to quickly find datasets of interest by entering keywords. **b** The introduction to drSMALL provides a brief overview of its functions and content. **c** This figure illustrates the relationships among the resident microbiota, molecules, host plants, and pathogens. **d** drSMALL offers four classification options: plants, microbes, molecules, and pathogens. By selecting these categories, users can access more detailed query interfaces

**Fig. 2:**
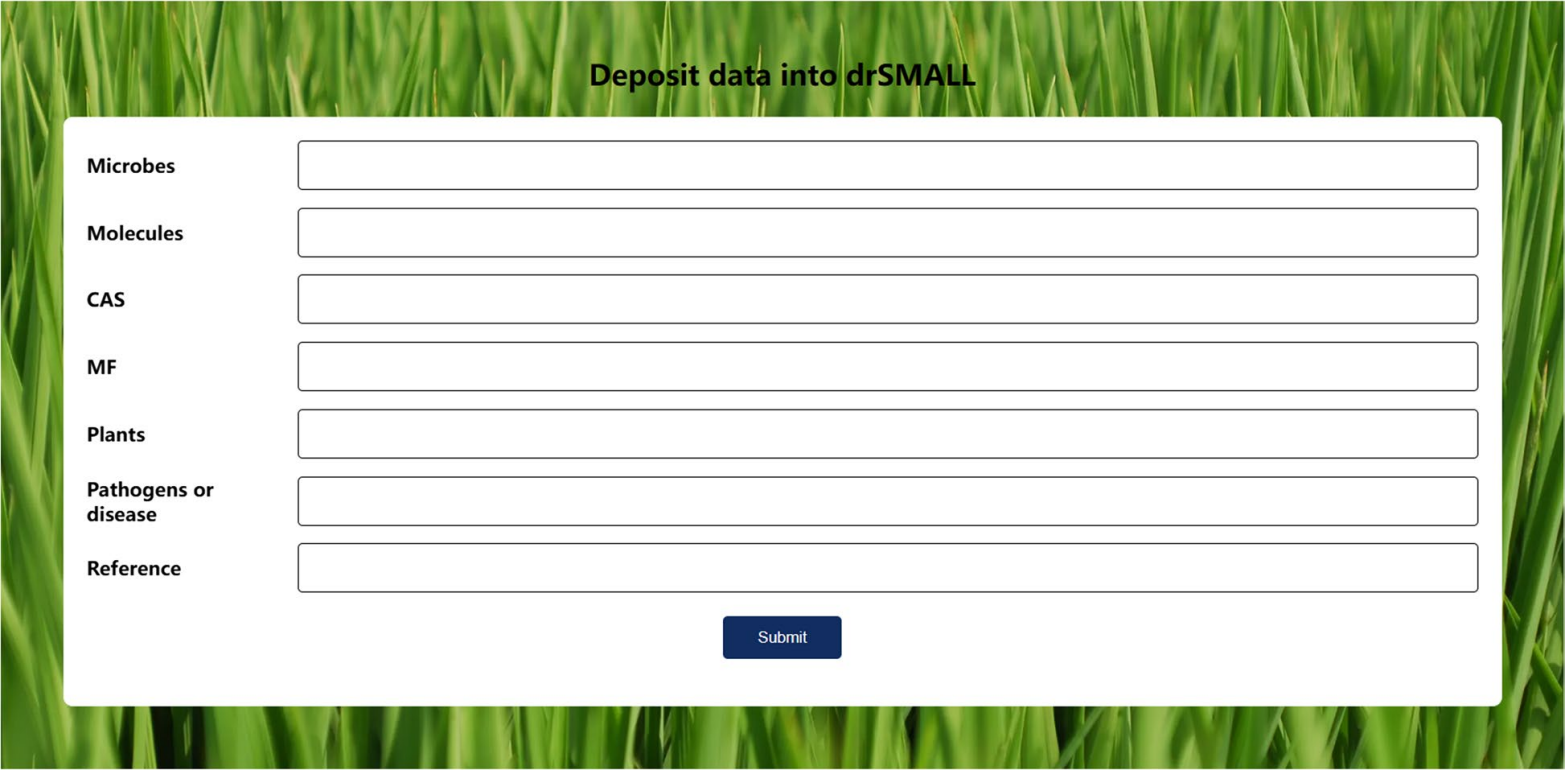
Submission interface of drSMALL. In the submission interface, users can contribute information about microbes, molecules, their CAS numbers, molecular formulas, host plants, targeted pathogens or diseases, and relevant references

To facilitate the elucidation and interrogation of small molecule functions, three core functionalities—Query, Classification Navigation, and Submission—were integrated into drSMALL (Fig. 1). The search box at the top of the page allows users to quickly find datasets of interest by entering keywords such as plants, microbes, molecules, or pathogens (Fig. 1a). Beneath the search box, a brief overview of the features and content of drSMALL is provided (Fig. 1b), allowing users to click for more detailed information. The central left section of the page displays the relationships among the resident microbiota, molecules, host plants, and pathogens (Fig. 1c), highlighting the core research subjects of the drSMALL database. On the right side, four categories of options are provided: plants, microbes, molecules, and pathogens (Fig. 1d). Users can access a more detailed query interface by clicking on these categories. For a more detailed understanding of the features and functions of drSMALL, users can refer to the "About" page. Furthermore, users are able to access the corresponding interface by clicking the "Submit", where they can provide relevant information to supplement the database (Fig. 2). Additionally, users who have any questions are invited to contact us at any time via the contact information provided on the "Contact" page. Through its clean and intuitive interface design and clear functional categorization, drSMALL offers a convenient and efficient platform that helps researchers better access small-molecule data related to the shaping of disease resistance.

## Discussion

Recent research has focused predominantly on the role of small molecules in enhancing disease resistance through the activation of plant immune responses and direct targeting of pathogens [7, 37–39]. However, the potential functions of these microbial small molecules in modulating other biological processes in plants remain largely elusive. Whether bioactive microbial small molecules possess additional biological activities essential for real agricultural practices, such as promoting plant growth, regulating plant metabolism, and enhancing tolerance to biotic and abiotic stresses [40], remains a significant knowledge gap. As a promising strategy, our database may serve as a library for screening potential multi-bioactivities hidden in the identified disease resistance-shaping microbial small molecules. Currently, while extensive information is available on pathogens and their associated plant hosts, data on symbiotic microbes and their plant hosts remain limited. This database addresses this gap by providing a novel, efficient approach for accessing essential information on key plant species and their closely associated symbiotic microbes.

Notably, some specific microbial species in the database are characterized by their capacity to secrete a variety of small molecules, suggesting that they may be reservoirs of bioactive molecules worthy of further exploration. Moreover, information regarding insufficiently studied crops, such as sorghum and radish, needs further attention and updating in the database. With the support of our evolving database, future research can explore the diverse functions of these small molecules in plant biology while also focusing on underexplored host plants and their resident microbiota.

## Conclusions and perspectives

drSMALL serves as a valuable informative platform for identifying small molecules produced by microorganisms and their role in shaping plant disease resistance. By integrating and systematizing data on microbial species, small molecules, and their host plants, drSMALL not only provides researchers with a convenient query tool but also lays a solid foundation for screening microbial molecules to gain a deeper understanding of their multifunctionality. The continuous updates and open-access nature of this database further enhance its value as an information hub for researchers in the field of plant–microbe interactions, supporting both fundamental and applied research. In summary, drSMALL offers a rapid pathway to identify microbial small molecules for exploring their previously unrecognized ecological and biological roles, thereby promoting the development of innovative strategies for sustainable agricultural production and global food security.

## Methods

### Data collection

To compile a database of plant-associated microbial small molecules, we conducted a comprehensive search via SciFinder (https://scifinder-n.cas.org/) with keywords such as “[Microbe]” AND “[Small Molecule]” AND “[Plant]” AND “[Disease Resistance]” up to October 2024. Here, “[Microbe]” was substituted with specific microbial taxa, such as “bacteria” and “fungi,” whereas “[Small Molecule]” included terms such as “secondary metabolites” and “natural organic compounds.” Only microbial small molecules with experimental evidence linking them to plant disease resistance or disease suppression were included in the database to ensure accuracy and consistency.

### Database construction and web implementation

The system is implemented as a web application to achieve interactive visualization and data retrieval. The front-end development is based on the Vue.js framework (https://vuejs.org/), enabling dynamic and user-friendly interfaces for data exploration. The back-end services are provided by the Spring Boot framework (https://spring.io/projects/spring-boot), which handles data collection, processing, and management efficiently. The open-source relational database management system MySQL (https://www.mysql.com/) is used to store and retrieve the curated drSMALL database.

## Data Availability

Not applicable.
